# The Perceptions of Nurses and Nursing Students Regarding Family Involvement in the Care of Hospitalized Adult Patients

**DOI:** 10.3390/nursrep11010013

**Published:** 2021-02-15

**Authors:** Faygah M. Shibily, Nada S. Aljohani, Yara M. Aljefri, Aisha S. Almutairi, Wassaif Z. Almutairi, Mashael A. Alhallafi, Fatmah Alsharif, Wedad Almutairi, Hanan Badr

**Affiliations:** 1 Critical Care Department, Faculty of Nursing, King Abdulaziz University, Jeddah 21589, Saudi Arabia; fshibily@kau.edu.sa; 2 Medical Surgical Department, Faculty of Nursing, King Abdulaziz University, Jeddah 21589, Saudi Arabia; naljohani0060@stu.kau.edu.sa (N.S.A.); yaljefri0001@stu.kau.edu.sa (Y.M.A.); aalmutairi0608@stu.kau.edu.sa (A.S.A.); walmutairi0054@stu.kau.edu.sa (W.Z.A.); malhalafy@stu.kau.edu.sa (M.A.A.); 3 Maternity and Child Department, Faculty of Nursing, King Abdulaziz University, Jeddah 21589, Saudi Arabia; walmutairi@kau.edu.sa (W.A.); habadr@kau.edu.sa (H.B.)

**Keywords:** perceptions held by nurses, patient-centered care, patient-centered nursing, family involvement in patient care

## Abstract

Over the past few decades, there have been concerns regarding the humanization of healthcare and the involvement of family members in patients’ hospital care. The attitudes of hospitals toward welcoming families in this respect have improved. In Arab culture, the main core of society is considered to be the family, not the individual. The objective behind involving family in patient care is to meet patients’ support needs. Consequently, this involvement affects nurses and their attitudes toward the importance of family involvement in patient care. Objectives: To describe nurses’ and nursing students’ perceptions of family involvement in the care of hospitalized adult patients in Saudi Arabia. Design: This study used a quantitative descriptive cross-sectional design. The data were collected using a convenience sampling survey via social media. Results: A total of 270 participants (staff and students) took part in this study, including 232 (85.9%) females and 38 (14.1%) males. Moreover, a high percentage of participants (78.8%) acknowledged that family presence strongly affected the improvement of the patient’s condition. However, 69.3% of participants thought that involving family members during special care processes or cardiopulmonary resuscitation (CPR) would be traumatizing for these individuals. Moreover, there was a significant diffidence between the attitudes of the nurses and nursing students toward family involvement and the number of years of employment (F = 3.60, *p* < 0.05). On the contrary, there were insignificant differences between the attitudes of the nurses and nursing students toward family involvement and their gender, nationality, age, education level, and years of work experience in Saudi Arabia (*p* > 0.05). Furthermore, the regression analysis showed a significant negative correlation between nurses’ years of employment and their support of family involvement in patient care (ß = −0.20, SE = 0.08, *t* = −2.70, *p* = 0.01). Conclusions: Nurses with more experience showed no support for family involvement in patient care. We have to consider the clinical barriers that affect nurses’ support for family involvement in patient-centered care, such as hospital polices, guidelines, and the model used for family-centered care integration in the hospital system to facilitate the interaction between healthcare providers and family members.

## 1. Background

Over the last few decades, concerns regarding the humanization of healthcare and the involvement of family members in hospital care have been observed. Nowadays, hospitals’ attitudes toward welcoming families in this context have improved [1]. In Arab culture, the main core of society is actually considered to be the family, not the individual. During one’s times of need, the expanded family structure can provide stability and physical and psychological support. Arab Muslim families prefer to focus on unity over a person’s individuality, because family unity is their priority [2]. The intention behind involving the family in a hospital setting is to meet the family members’ needs for support and information and to provide them the opportunity to be close to the patient. In addition, family participation in hospital care leads to better experiences and enhances the outcomes for the patients [3,4,5]. Nurses meet families in all healthcare contexts, and the value of these meetings affects the nurses and their attitudes toward family involvement in patient care [6]. Therefore, the aim of our study was to assess nurses’ and nursing students’ perceptions of family involvement in patient care.

The family members of patients play an essential role in providing care for them. This includes being with them for the majority of the time and supporting and assisting with their activities [5]. Nurses sometimes have mixed feelings about the involvement of family members; they may view the relatives as either a benefit or a burden. From a nurse’s point of view, involving family members may lead to additional work; however, these family members can also play an important role in providing care to the patient [7]. The literature on the subject of family involvement includes many mentions of both positive and negative attitudes of nurses in this respect [8]. The present study focused on nurses’ perceptions of family presence and involvement in the hospital care of adult patients in specific contexts, including daily care, cardiopulmonary resuscitation (CPR), and special care.

Nurses’ acceptance of the significance of family with regard to providing care to patients differs depending on the context of the care [9]. For example, nurses who work in obstetrics departments have been reported to be more accepting of the help of the patient’s family members than those working in the emergency department [9,10]. One study found that nurses believe that families are a source of integrated knowledge of the health status of the patients in question and that they can help improve their relative’s pain management [1]. The same study also highlighted that nurses respect patients’ families and attempt to alleviate their concerns [1]. On the contrary, some nurses are ready to engage with the family and accept their assistance only if the relatives work to make the nurse’s work easier. Nurses also believe that the families might misunderstand the information provided to them [1].

Additionally, nurses tend to prioritize patients’ desires and rights over their families’ preferences. This has caused their engagement with hospitalized patients’ families to be limited when taking care of said patients [1]. Furthermore, another study emphasized that nurses are hesitant to involve families in the process of care-giving, even when the family members convey their willingness to engage in the care of their loved ones [11]. The desire to play even a small role in taking care of their relatives is something that family members have expressed for a long time. In addition, nurses have realized how beneficial it is for a patient’s family to be involved in the provision of their care; guiding family members with regard to caring for a patient benefits both the nurse and the family [11]. According to another study, nurses state that, for family members to be involved in patient care, they need to meet three criteria: they have to be willing to engage, emotionally stable, and cooperative [12].

One study indicated the importance of educational and training programs with regard to family-centered care in nursing practice [13]. The majority of the participants who had higher levels of education and had undertaken a training program on family-centered care stated that they felt in control and more skilled at their work [13]. A few studies have explained the attitudes of nurses and nursing students toward the involvement of family in the context of daily care and CPR [14,15]. In addition, previous research has focused primarily on limited aspects and have not paid heed to family participation in hospital settings [8,9,10,11,12,13,14,15,16]. The current study aimed to describe nurses’ and nursing students’ perceptions of family involvement in the care of hospitalized adult patients.

## 2. Methods

### 2.1. Study Design

A quantitative descriptive cross-sectional approach was used in this study. The sample size was calculated using G*power 3.1.9.7 software (effect size = 0.3; power = 0.95; alpha = 0.05, two-tailed). The study conducted by Al-Mutair et al. in 2012 [10] was used as a reference to calculate the sample size.

### 2.2. Setting

Data were collected using convenience sampling of nursing employees and nursing students with different levels of education via social media platforms such as Twitter, WhatsApp, and Facebook. The data were collected from a university and a university hospital located in the western region of Saudi Arabia. The online data collection enabled easier access to the target population during the coronavirus disease 2019 (COVID-19) pandemic while maintaining the safety of everyone involved.

### 2.3. Ethical Considerations

Ethical approval was obtained from the faculty of nursing of King Abdalaziz University in Jeddah, Kingdom of Saudi Arabia. Participation was optional for both nurses and nursing students. We explained the aim of our study through the survey, and the participants acknowledged that they understood that all of their information would be confidential and used solely for the purpose of this study. Subsequently, those who agreed to participate in the survey moved forward to the question stage, whereas those who disagreed were excluded from the study.

### 2.4. Inclusion and Exclusion Criteria

To meet the inclusion criteria for this study, participants had to be nurses or nursing students who were able to speak and read English. The students in the Saudi Nursing Program were expected to have completed five years of school. The students start carrying out practical clinicals and begin taking care of patients in their third year. Therefore, first- and second-year students were excluded from this study, as they would not have had any clinical experience with patients. Moreover, in the fifth year of the nursing program, it is mandatory for nursing students to practice and work in a hospital for one year, serving as an intern in different departments, before graduating from school.

The link to the questionnaire was shared online via social media (WhatsApp, Facebook, and Twitter), and the option to participate was entirely dependent on the defined inclusion and exclusion criteria in order to guarantee that only the target population of this study was included. If participants checked one of the exclusion criteria, they were not able to complete the survey.

### 2.5. Questionnaire

The data were collected using a 2014 questionnaire developed by Al-Mutair et al. [10]. The questionnaire was previously approved by the Nursing College of King Abdulaziz University. In addition, the original author had already tested the questionnaire’s reliability and validity by consulting 12 professionals (critical nurses, nurse managers, nurse academics, and statistics consultants). Based on the feedback of these professionals, the authors were able to adjust and modify the questions. After testing the questionnaire’s validity and reliability, seven staff members (nurses, physicians, and respiratory therapists) from the intensive care unit (ICU) were asked to test the questionnaire items to confirm that the they were easy to comprehend.

The questionnaire was in English and contained two sections. The first section involved sociodemographic data, such as age, gender, level of education, years of employment, and years of work experience in Saudi Arabia. The second section was associated with nurses’ and nursing students’ attitudes toward involving the family of patients in their daily care, CPR, and other special care. Furthermore, the second section was divided into two parts: the first part included 14 items regarding the attitudes of nurses and nursing students toward involving families in daily care, and the second part consisted of eight items regarding their attitudes toward the presence of relatives during CPR or other special care. The answers to the items were recorded on a five-point Likert-type scale, where the possible responses were (1) strongly disagree, (2) disagree, (3) uncertain, (4) agree, and (5) strongly agree.

### 2.6. Data Collection

The researchers received permission to use the questionnaire created by Al-Mutair et al. from the original author on 25 February 2020. Then, the questionnaire was sent to the participants over the internet using Google Forms. It was distributed via social media such as WhatsApp, Twitter, and Facebook. The form was open from 1 to 30 March 2020. Once a sufficient number of participants had completed the survey, data collection was ceased, and we began analyzing the gathered data.

### 2.7. Statistical Methods

The data were analyzed using (SPSS software) (IBM, Inc., Chicago, IL, USA) version 26. 

The frequencies, percentages, means, and standard deviations were calculated for the questionnaire items and sociodemographic factors. The relationships between the variables were tested based on the data; a Pearson’s correlation test, an independent *t*-test, a one-way analysis of variance (ANOVA) (with the consideration of using posthoc comparisons using Tukey’s honest significant difference test when differences occurred), and linear regression were conducted. A *p*-value of <0.05 was considered statistically significant.

## 3. Results

### 3.1. Participants

There were a total of 270 participants (staff and students) in this study, including 232 (85.9%) females and 38 (14.1%) males. The majority of the participants were young: 196 (72.6%) were 20–25 years old. Additionally, 186 (68.8%) were students, and 154 (57%) had no work experience in Saudi Arabia (see Table 1).

### 3.2. Attitudes of Nurses toward Family Involvement in Daily Care and Special Care

Al-Mutair’s tool was designed to use a Likert scale [10]. For the most part, we used it as is without making any changes. However, in Section 3.2, Section 3.3 and Section 3.4, we combined “agree” and “strongly agree” into one response and “disagree” and “strongly disagree” into one response. The data analysis demonstrated that the majority of the participants agreed with most of the questionnaire items. In total, 79.3% of the nurses and nursing students supported family involvement during daily care. Furthermore, 43% of them supported the presence of family during CPR and other special care.

### 3.3. Roles of Family

The majority of the participants (83.8%) believed that family presence allowed the members of the family to remain informed about the patient’s status. A large percentage (78.8%) acknowledged that family presence strongly affected the improvement of the patient’s condition. Additionally, 76.7% believed that family involvement in patient care improved the communication between nurses and relatives and gave families a sense of confidence in the care being provided. Moreover, 74.4% of the participants believed that being involved during the provision of care gave the family clear evidence that the patient was receiving the best care possible. Furthermore, 71.1% of participants indicated that when the presence of a family was permitted, the concerns and anxiety of the family members were reduced. In addition, 43% of the respondents believed that it was easy to manage critical family situations when the family was with the patient, whereas 32.6% of them disagreed with this statement. Moreover, 38.9% of the respondents reported that the presence of family during the CPR process or an invasive procedure helped them obtain the patient’s health history more quickly.

### 3.4. The Impact of Family Presence

Two-thirds (66.6%) of the participants said that the presence of the patient’s family made them provide more thorough care to the patient. Furthermore, 50% of the participants reported that their manner of working would not be influenced by the presence of relatives. Of those surveyed, 38.9% did not consider family presence to be a source of stress for them. Overall, 45.9% of the participants did not feel that their professional duties prevented them from involving the patient’s family in their loved one’s care.

### 3.5. Preparedness for Family Involvement through Training Programs

Our data revealed that 53.3% of the respondents believed that they were adequately trained in the identification of family necessities. Additionally, 51.4% of the respondents said that they had received the appropriate training with regard to family-centered care.

### 3.6. Presence of Family Members during Resuscitation and Invasive Procedures

Of the nurses and nursing students surveyed, 69.3% thought that involving family members during special care processes or CPR would be traumatizing for these individuals. However, 34.5% thought that family members had the right to stay at their loved one’s bedside pending resuscitation or an invasive procedure, while 56% thought that family members should provide informed consent in such situations. Over half (54.8%) of the respondents stated that it was the duty of the hospital to provide the relevant guidelines to enhance family members’ participation and to offer them the choice to be present during resuscitation or an invasive procedure. Furthermore, 74.1% of the respondents said that it was the duty of the hospital to develop special programs for training nurses to support family members when they were present during resuscitation or invasive procedures.

### 3.7. The Association between the Attitudes of Nurses and Nursing Students toward Family Involvement during Daily and Special Care and Their Sociodemographic Factors

Table 2 illustrates the association between the nurses’ and nursing students’ sociodemographic factors and their attitudes toward family involvement during daily and special care. A one-way ANOVA was conducted. The only significant result found was associated with the attitudes of the nurses and nursing students toward family involvement and the number of years of employment (F = 3.60, *p* < 0.05). Posthoc comparisons made using Tukey’s honest significance test indicated that the mean score for those who had worked for more than 10 years (mean = 3.15, standard deviation = 0.94) was significantly different from those who had not (mean = 3.59, standard deviation = 0.56). On the contrary, there were insignificant differences between the attitudes of the nurses and nursing students toward family involvement and their gender, nationality, age, education level, and years of work experience in Saudi Arabia (*p* > 0.05).

#### Regression Analysis

As shown in Table 3, linear regression was used to test the effect of the sociodemographic factors on the attitudes of nurses and nursing students toward family involvement during routine and special care. The significant value of the path estimation (β) was examined based on the *t*-value (*p* < 0.05). R^2^ is a function of the influence of the independent variable on the dependent variable, so the R^2^ of the independent variables (predictors variables) had a value of 0.025. This means that only 2.5% of the influence was caused by independent variables (predictor variables). However, only “years of employment” presented a significant effect (β = −0.20, *t* = −2.70, *p* < 0.05).

## 4. Discussion

To the best of our knowledge, our study is one of the first to focus on the perception of nurses and nursing students in Saudi Arabia regarding the involvement of family in the care of their patients in a hospital setting.

In this study, nurses and nursing students reported having a positive perception of family involvement in most of the questionnaire items. Two previous studies also found that nurses had a positive perception of family participation and did not see it as a hindrance to their clinical performance [6,9]. According to our results, the majority of the participants believed that the presence of family members allowed these relatives to remain informed about the status of the patient in question and, in addition, affected the improvement of the patient’s condition. A previous study stated that providing frequent updates about patients’ conditions and prognoses gave families a sense of control and awareness that facilitated their involvement in the patient’s care, giving them a better experience and enhancing the final outcomes [5]. The majority of the participants in this study indicated that, when a patient’s family was permitted to remain present during the provision of care, the family members’ concerns and anxiety were reduced. These results were similar to those of a previous study, which stated that giving family members a chance to be around the patient helped decrease both the family’s and the patient’s fears and anxiety [11].

Most of the participants in the current study agreed that when the patient’s family was present, it made the care being provided better. Furthermore, half of the participants agreed that the presence of family members did not affect their clinical performance. Participants also felt that their numerous professional duties did not prevent them from involving family members in the care of the patient. There was a disparity among the participants with regard to the belief that a family’s presence during treatment was considered a source of stress. It seems that certain participants believed the validity of this to be dependent on the place and situation. In addition, a previous study proved that the training program about family-centered care that is a part of nursing education enhanced the nursing practices associated with the inclusion of family members in patient care [15].

More than half of the respondents in this study said that they were adequately trained in the identification of family necessities and that they received appropriate training on family-centered care. Most nurses and nursing students believed that involving family members during invasive procedures or resuscitation could prove to be traumatizing for them [15]. Some of the nurses believed that the family had the right to be present during CPR processes or other invasive procedures, while certain respondents stated that it was the duty of the hospital to provide guidelines to enhance family participation and to offer families the choice to remain present as the patient undergoes CPR or any invasive procedures [15,16]. In addition, some believed that it was the duty of the hospital to develop special programs for training nurses to support family members who were present during CPR or invasive procedures [15,16]. One study revealed that nurses feared family members being psychologically damaged and causing distractions during CPR processes [17]. Another study emphasized that most nurses who were confident in their abilities exhibited a supportive attitude toward the presence of family during CPR, whereas nurses who were less confident in this regard found that this influenced their attitude toward the involvement of family [13]. Further, a different study revealed that the majority of healthcare professionals and nurses did not prefer family members being present during resuscitation or other invasive procedures [10]. Professionals and nurses feared that witnessing a resuscitation might cause family members to become emotionally disturbed. Healthcare providers and nurses expressed that if family members were to be present during the resuscitation of their loved ones, they should be required to provide written informed consent. The vast majority suggested that there should be guidelines to support family involvement in the treatment of patients [9]. Yet another study stated similar results; that is, the majority of healthcare professionals, including nurses, did not prefer the presence of family during CPR processes for several reasons [10]. They considered it to be traumatic for the family to witness the resuscitation or procedure of a loved one. However, they were generally accepting of family presence during CPR if the family was fully informed and supported by a well-trained grief team member. In general, nurses had a more positive attitude than physicians regarding family members being present during the performance of CPR [14].

Most of the participants in the present study were nursing students. Those with no nursing employment experience comprised 68.8% of the total participants. These participants were supportive of involving patients’ family members in the process of their treatment. In contrast, Blöndal et al. [7] determined that younger, less experienced nurses in the field (and those with no experience) showed no interest in the family’s participation in patient care. Another study found that young and inexperienced nurses considered the family a load on their shoulders and believed that they needed more support to implement family-centered care. In recent years, the focus of nursing programs on family-centered care has increased, therefore increasing students’ awareness of and acceptance toward family involvement in patient care.

Moreover, there was a significant relationship between the number of years of employment and the attitudes of the nurses and nursing students toward family involvement (F = 3.60, *p* < 0.05). The nurses who had more years of work experience were more opposed to involving the family in the treatment of their patient. A study showed that most registered nurses in primary healthcare accepted family involvement in patient care, while, overall, hospital nurses were less accepting than primary healthcare nurses of family involvement during the treatment of patients [6]. Nurses in primary care do not have the workload of those who work in hospital units, especially in the critical care departments, because the patients involved have a considerable number of needs. Furthermore, another study yielded similar results to this study, with the researchers recommending that nursing programs should incorporate the involvement of family members in patient care in the program curriculum [18].

## 5. Limitations

We originally planned to collect the required data using physical or electronic forms during shift changes for the professionals and during lectures for the students. However, the COVID-19 pandemic hit Saudi Arabia extremely hard, and travel became restricted. As a result, we changed our method and, instead, collected the data via social media in order to reach nursing students and staff within the time frame set by IRB. This is a major bias in our study and affected our data. The majority of our participants were nursing students, while the minority were nursing staff. We emailed the head nurses of the hospital. However, we believe that they were occupied with the huge number of COVID-19 cases that the hospital was receiving at the time. Notably, we did not evaluate any data prior to ceasing data collection. We discovered the notable difference between the numbers of nursing students and staff only after the data collection period had ended, during the data analysis stage. The fact that only a few employed nurses participated in this study limits the generalizability of its results.

Moreover, another limitation of our study was that the data were collected in terms of categorical and ordinal parameters, such as age, which led to us losing out on the richness offered by continuous data and to the generalizability of this study being limited.

## 6. Study Implications

It is important for future researchers to collect qualitative data that can provide more in-depth information regarding why experienced nurses prefer not to involve family members in patient care and whether the culture or the system does not support the concept of involving family members in this manner. Moreover, the challenges involved with the integration of family members into patient care should be assessed and examined.

## 7. Conclusions

Nurses with clinical experience in the western region of Saudi Arabia showed no acceptance toward the idea of involving family members in patient care. It is important to assess the hindrances that affected the nurses’ support for center care, such as hospital polices and guidelines and the model of family- and patient-centered care integration used in the hospital system that could facilitate the interaction between nurses and family members.

## Figures and Tables

**Table 1:** Sociodemographic details of the study’s participants.

Factor		*n*	%
Gender	Male	38	14.07
	Female	232	85.93
Nationality	Saudi	251	92.96
	Foreigners	19	7.04
Age	20–25	196	72.59
	26–30	39	14.44
	31–35	24	8.89
	36+	11	4.07
Education level	Third-year nursing student	70	25.93
	Fourth-year nursing student	84	31.11
	Fifth year nursing student (nursing intern)	32	11.85
	Diploma in nursing	11	4.07
	Bachelor’s degree in nursing	57	21.11
	Master’s degree in nursing	10	3.70
	Ph.D. in nursing	6	2.22
Years of employment	None	183	67.78
	<1 year	17	6.30
	1–5 years	35	12.96
	6–10 years	15	5.56
	>10 years	20	7.41
	None	154	57.04
Years of work experience in Saudi Arabia	<1 year	45	16.67
	1–5 years	40	14.81
	6–10 years	12	4.44
	>10 years	19	7.04

Total *n* = 270. This table shows descriptive statistics: frequency (*n*) and percentage (%).

**Table 2:** The associations between the attitudes of nurses and nursing students toward family involvement during routine and special care and their sociodemographic factors (*n* = 270).

Factor	Levels	M	SD	Statistical Test	*p*-Value
**Gender**	Male	3.44	0.68	*t* = −0.82	0.41
	Female	3.53	0.58		
**Nationality**	Saudi	3.52	0.59	*t* = 0.99	0.32
	Non-Saudi	3.39	0.58		
**Age**	20–25	3.57	0.54	F = 1.89	0.13
	26–30	3.38	0.54		
	31–35	3.36	0.87		
	36+	3.40	0.85		
**Education level**	Third-year nursing student	3.54	0.60	F = 1.34	0.24
	Fourth-year nursing student	3.62	0.54		
	Nursing intern	3.45	0.73		
	Diploma in nursing	3.55	0.57		
	Bachelor’s degree in nursing	3.38	0.45		
	Master’s degree in nursing	3.58	1.03		
	Ph.D. in nursing	3.23	0.61		
**Years of employment**	None	3.59	0.56	F = 3.60	0.007 **
	<1 year	3.37	0.58		
	1–5 years	3.37	0.49		
	6–10 years	3.63	0.41		
	>10 years	3.15	0.94		
	None	3.59	0.57		
**Years of work experience in Saudi Arabia**	<1 year	3.39	0.70	F = 1.76	0.14
	1–5 years	3.45	0.53		
	6–10 years	3.61	0.45		
	>10 years	3.33	0.68		

*t*, *t*−test; F, ANOVA test; *p*−value, significance level; M, mean, SD, standard deviation ** *p* ≤ 0.05.

**Table 3:** Predictors of the attitudes of nurses and nursing students toward family involvement during routine and special care (*n* = 270).

Predictor Variables	ß	SE-b	Beta	*t*	*p*	95% CI	
						Lower	Upper
Gender	0.01	0.11	0.01	0.09	0.93	−0.20	0.22
Nationality	−0.09	0.14	−0.04	−0.64	0.52	−0.37	0.19
Age	0.05	0.08	0.07	0.58	0.56	−0.11	0.21
Education level	−0.01	0.04	−0.04	−0.35	0.72	−0.09	0.06
Years of employment	−0.20	0.08	−0.44	−2.70	0.01 **	−0.35	−0.06
Years of work experience in Saudi Arabia	0.13	0.07	0.28	1.80	0.07	−0.01	0.28

The dependent variable was the attitudes of the nurses and nursing students toward family involvement during routine and special care. CI, confidence interval; *p*-value, significance level; SE-b, standard error; ß, unstandardized coefficient. ** *p* ≤ 0.05; R^2^ = 0.047; adjusted R^2^ = 0.025.

## Data Availability

The data is available from the authors on request.
